# Comparison of clinical effects between microscopic surgery and conventional surgery in children with penile hypospadias and difference analysis of postoperative urodynamics

**DOI:** 10.12669/pjms.39.5.6986

**Published:** 2023

**Authors:** Shi-lei Guo, Wei Zheng, Xiao-qing Shi, Bo-song Zhang, Jie Wang

**Affiliations:** 1 Shi-lei Guo, Department of Urology Surgery, Beijing Children’s Hospital Affiliated to Capital Medical University Baoding Hospital, Baoding 071000, Hebei, China; 2 Wei Zheng, Department of Urology Surgery, Beijing Children’s Hospital Affiliated to Capital Medical University Baoding Hospital, Baoding 071000, Hebei, China; 3 Xiao-qing Shi, Department of Urology Surgery, Beijing Children’s Hospital Affiliated to Capital Medical University Baoding Hospital, Baoding 071000, Hebei, China; 4 Bo-song Zhang, Department of Urology Surgery, Beijing Children’s Hospital Affiliated to Capital Medical University Baoding Hospital, Baoding 071000, Hebei, China; 5 Jie Wang, Department of Urology Surgery, Beijing Children’s Hospital Affiliated to Capital Medical University Baoding Hospital, Baoding 071000, Hebei, China

**Keywords:** Penile hypospadias, Microscopic surgery, Clinical effect, Urodynamics

## Abstract

**Objective:**

To compare and analyze the clinical effects of microscopic surgery and conventional surgery in children with penile hypospadias and the differences in postoperative urodynamic indexes.

**Methods:**

It was a clinical comparative study. A total of 80 children with penile hypospadias admitted to Beijing Children’s Hospital Affiliated to Capital Medical University Baoding Hospital from July 2018 to September 2022 were selected and randomly divided into two group. The experimental group were treated with microscopic urethroplasty, while the control group were treated with traditional urethroplasty. The operative effect, operation time, total intraoperative blood loss, postoperative hospital stay and incidence of surgical complications were compared and analyzed between the two groups. All the children were followed up for two years, and the changes in urodynamic parameters including maximum urine flow rate (Qmax), average urine flow rate (Qavc), urine flow time (FT), peak time (TQmax) and residual urine (PVR) were compared before, two weeks after, six months after and two years after surgery.

**Results:**

The efficacy of the experimental group was significantly higher than that of the control group (p=0.013). The intraoperative blood loss and postoperative hospital stay in the experimental group were significantly better than those in the control group (p=0.000). The incidences of urinary leakage and urethral stricture in the experimental group were lower than those in the control group (p<0.05). The Qmax level in the experimental group was higher than that in the control group at six months and two years after surgery, while the FT level was lower than that of the control group (p<0.05).

**Conclusion:**

Microscopic surgery is a method with significant clinical value in the treatment of penile hypospadias.

## INTRODUCTION

Hypospadias, one of the most common congenital abnormalities in males^1^, is characterized by ectopic urethral orifice, penile curvature and headscarf-like changes of the dorsal foreskin.^2^ About 70% of children with hypospadias are penile.^3^ When repairing hypospadias, normal appearance and function are usually expected.^4^ A number of surgical approaches are available for hypospadias. At present, there are many kinds of urethroplasty applied clinically, but the general principle is to build a new urethra between the external urethra and the penis head and lay a multilayer tissue barrier between the new urethral canal and the skin to prevent urinary leakage.^5^

After surgery on hypospadias, the most common postoperative complications include postoperative urinary fistula, urethral stricture, and unsatisfactory appearance. With the continuous development of science and technology, microsurgical techniques have achieved initial success in the repair of hypospadias. Microscopic techniques can be used to perform more precise tissue manipulations and clearly observe the direction of blood vessels, thereby ensuring good blood supply to the covering tissue, improving the success rate of surgery, and reducing the incidence of postoperative complications.^6^

In this study, the advantages of using microscopy in urethroplasty, the incidence of adverse reactions and postoperative complications were mainly evaluated, and the urodynamic parameters were used to make a comparative analysis between the microscopic surgery group and the conventional surgery group. Based on the changes in urodynamic indexes, we can provide a basis for early diagnosis and prediction of postoperative urethral stricture.

## METHODS

It was a clinical comparative study. A total of 80 children with penile hypospadias admitted to Beijing Children’s Hospital Affiliated to Capital Medical University Baoding Hospital from July 2018 to September 2022 were selected and randomly divided into two groups: the experimental group and control group, with 40 cases in each group. Patient data comes from the electronic medical record system in our hospital. Patients in the experimental group were treated with microscopic urethroplasty, while those in the control group were treated with traditional urethroplasty. No significant difference was observed in the comparison of the general data of the two groups (p>0.05), which was comparable (Table-I). The study was approved by the Institutional Ethics Committee of Baoding Children’s Hospital (No.:2021010; date: August 2, 2021), and written informed consent was obtained from all participants.

**Table-I:** Comparative analysis of the general data of the two groups (*χ̅*±*S*) n=40.

Index	Experimental group	Control group	t/χ^2^	p
Age	6.55±1.58	6.85±1.75	0.804	0.424
***Urethral orifice position***			0.731	0.694
Distal type (%)	22 (55.00%)	25 (62.50%)		
Central type (%)	13 (32.50%)	12 (30.00%)		
Proximal type (%)	5 (12.50%)	3 (7.50%)		
***Penis curvature***			0.251	0.617
Yes (%)	10 (25.00%)	12 (30.00%)		
No (%)	30 (75.00%)	28 (70.00%)		

p>0.05.

### Inclusion criteria:

- Children who met the diagnostic criteria of penile hypospadias and have surgical indications.^7^
- Children with hypospadias without or only mild hypospadias.
- Children aged ≤ 12 years.
- Children with complete clinical data.
- Children whose families agreed to accept the study and signed the informed consent form.
- Children with no obvious symptoms of the mental nervous system, had good treatment compliance and able to cooperate with the completion of the study.

### Exclusion criteria:

- Children who underwent repair surgery again due to complications after stage I surgery.
- Children whose urethral orifice was located at the penis head, coronal sulcus or the junction of penis and scrotum.
- Children who are unable to tolerate surgery because of dysfunction of important organs such as heart and lung.
- Children with coagulation disorders or unable to operate for other reasons.

### Surgical methods

Children in the experimental group were treated by microscopic surgery. All the children underwent surgery under general anesthesia. They were connected to a microscope surgical system, the head of the penis was fixed with 4-0 silk sutures with proper traction. A U-shaped incision was made below the urethral orifice. The size of the flap can keep the reconstructed urethra with a certain width. The foreskin was incised to Buck’s fascia along 0.5cm below the coronary sulcus, and the skin and subcutaneous fascia were released to straighten the penis. With an appropriate indwelling silicone catheter, the urethral plate edge flap was sutured intermittently with 6-0 Vicryl suture under the microscope, starting from the proximal urethral orifice to the head of the penis.

The newly-built urethral opening tunneled through the penis head and was intermittently sutured at the normal urethral opening of the penis head, and the indwelled catheter in the new urethra was properly cut. Part of the tunica muscularis dorsalis of the penis was freed or an appropriate size of tunica vaginalis was taken, and the pedicle passed through the root of the scrotum and covered the newly-built urethra. After that, the dorsal foreskin was longitudinally cut and transferred to the ventral side to wrap around the penis, and the wound was closed and bandaged with sterile gauze. Children in the control group were operated by the traditional operation method, traditional surgical methods, and the surgical procedures and steps were the same as those in the experimental group. All operations were performed by the same group of doctors, and all the children were followed up for two years.

### Observation indexes:

### Surgery-related indexes

The indexes of the operation time, total intraoperative blood loss, and postoperative hospital stay between the two groups were compared and analyzed. (2) ***The surgical effects:***

### Markedly effective

All the symptoms of the children disappeared, and they could urinate on their own;

### Effective

children showed relieved symptoms and could stand to urinate after treatment, but had symptoms of pain and discomfort;

### Invalid

No improvement in the symptoms of the children. Total effective rate = (Markedly effective+effective)/total cases × 100%^8^;

### Comparative analysis of surgical complications

Comparative analysis of urinary fistula, urethral stricture and unsatisfactory penile appearance after two weeks and six months after surgery;

### Changes of urodynamic indexes

The changes of urinary dynamic parameters including Qmax, Qavc, FT, TQmax and PVR were compared before, two weeks after, six months after and two years after surgery.

### Statistical analysis

All the data in this study were statistically analyzed by SPSS 20.0 software, and the measurement data were expressed in (*χ̅*±*S*). Two independent samples t test was used to analyze the data between the experimental group and the control group. The c^2^ test was used for rate comparison. P<0.05 indicates a statistically significant difference.

## RESULTS

The blood loss and postoperative hospital stay of the experimental group were significantly better than those of the control group (p=0.000) (Table-II). The effective rate of the experimental group was 97.50%, which was significantly higher than 80.00% of the control group (p=0.013) (Table-III). The incidences of urinary leakage (p=0.040) and urethral stricture (p=0.048) in the experimental group were lower than those in the control group, with a statistically significant difference (Table-IV).

**Table-II:** Comparative analysis of the surgical conditions of the two groups (*χ̅*±*S*) n=40.

Group	Operation time (min)	Blood loss (ml)*	Postoperative hospital stay (d)*
Experimental group	124.45±7.59	20.35±3.25	10.35±0.77
Control group	125.68±4.85	24.90±4.48	13.55±1.34
t/c^2^	0.860	5.199	13.106
p	0.392	0.000	0.000

* p<0.05.

**Table-III:** Comparative analysis of surgical effect indexes of the two groups (*χ̅*±*S*) n=40.

Group	Markedly effective	Effective	Invalid	Total effective rate *
Experimental group	37	2	1	39 (97.50%)
Control group	31	1	8	32 (80.00%)
c^2^				6.135
p				0.013

* p<0.05.

Before surgery, no significant difference was observed in Qmax, PVR, Qavc, FT, and TQmax of the two groups (p>0.05). The Qmax level in the experimental group was higher than that in the control group at six months and two years after surgery, while the FT level was lower than that of the control group (p<0.05). Two years after surgery, the level of Qavc in the experimental group was higher than that in the control group, while the TQmax level was lower than that in the control group (p<0.05) (Table-V).

**Table-IV:** Comparative analysis of the incidence of complications between the two groups at 2 weeks and 6 months after surgery (*χ̅*±*S*) n=40.

Group	Urine leakage (%)		Urethral stenosis (%)			Unsatisfactory appearance (%)
*Time*	2 weeks after surgery*	6 months after surgery*	*2 weeks after surgery*	6 months after surgery*	*2 weeks after surgery*	*6 months after surgery*
Experimental group	0 (0.00%)	0 (0.00%)	0 (0.00%)	1 (2.50%)	2 (5.00%)	2 (5.00%)
Control group	4 (10.00%)	4 (10.00%)	3 (7.50%)	6 (15.00%)	3 (7.50%)	3 (7.50%)
c^ 2^	4.211	4.211	3.117	3.914	0.213	0.213
p	0.040	0.041	0.077	0.048	0.644	0.644

* p<0.05.

**Table-V:** Comparative analysis of urodynamic indexes between the two groups before and after surgery.

Index	Group	Before surgery	2 weeks after surgery	6 months after surgery *	2 years after surgery *
Qmax (ml/s)	Experimental group	9.52±3.54	6.32±3.30	8.67±3.08	9.53±3.02
	Control group	9.26±3.38	6.26±3.13	7.15±3.00	8.09±2.98
	t	0.325	0.082	2.238	2.147
	p	0.739	0.935	0.028	0.035
Qavc (ml/s)	Experimental group	6.42±2.19	4.93±2.09	6.49±2.03	6.87±2.08
	Control group	6.46±2.17	4.65±2.07	5.62±2.02	5.77±2.06
	t	0.091	0.608	1.920	2.388
	p	0.928	0.545	0.059	0.019
FT (s)	Experimental group	29.03±7.24	31.06±7.63	23.81±7.32	21.41±7.16
	Control group	29.25±7.01	32.25±7.72	28.63±7.49	27.23±7.40
	t	0.138	0.692	2.911	3.572
	p	0.891	0.491	0.005	0.001
TQmax (s)	Experimental group	14.51±9.12	11.92±9.06	10.34±8.79	5.99±5.81
	Control group	13.18±8.53	11.59±8.49	10.04±8.24	9.43±8.19
	t	0.675	0.170	0.157	2.164
	p	0.501	0.866	0.876	0.034
PVR (ml)	Experimental group	13.43±5.49	17.93±5.41	13.04±5.27	12.07±5.16
	Control group	12.75±5.13	18.05±4.85	13.32±5.01	12.62±4.75
	t	0.573	0.104	0.241	0.493
	p	0.569	0.917	0.810	0.624

* p<0.05.

## DISCUSSION

It was confirmed in our study the effective rate of the experimental group was significantly higher than that of the control group. The intraoperative blood loss and postoperative hospital stay in the experimental group were significantly better than those in the control group. The incidence of urinary leakage and urethral stricture in the experimental group were lower than those in the control group, with a statistically significant difference. The Qmax level in the experimental group was higher than that in the control group at six months and two years after surgery, while the FT level was lower than that of the control group. Two years after surgery, the level of Qavc in the experimental group was higher than that in the control group, while the TQmax level was lower than that in the control group. The conclusion of this study provides a new clinical reference for the treatment of penile hypospadias with microsurgery.

Hypospadias, one of the most common congenital genital malformations in male children, often occurs in infants and young children.^9^ Clinically, orthopedic surgery is regarded as a gold standard in the treatment of hypospadias.^10^ In terms of the most common complications of hypospadias repair, urinary fistula, postoperative neourethral stricture and unsatisfactory appearance were prominent. Especially urine leakage, repeated repair brings heavy financial burden and psychological pressure to the families of children^11^ microscopic surgery is a rapidly developing surgical technique in recent years, which can enlarge tissues and blood vessels by corresponding times via a surgical microscope, providing clearer surgical vision and ensuring more precise operation.^12^

Clinically, the anatomical structures involved in hypospadias surgery are limited to the penis and scrotum, small and subtle parts. For this reason, a well-illuminated and enlarged surgical field is essential for optimal outcomes.^13^ It was considered by Snodgrass et al.^14^ that complete blood supply and local minor tissue injury are the key to reducing postoperative complications. El-Karamany et al.^15^ concluded that microsurgical techniques could reduce postoperative pain, hospital stay and dressing change time, and even if a urethral stent was not used postoperatively, infants with distal hypospadias had a low complication rate without compromised short-term functional and aesthetic outcomes.

Urethral duct stenosis and urinary leakage are common complications after surgery for hypospadias, which can be attributed to inflammation, flap ischemia, scar healing, etc. Effective anti-inflammatory and prepuce skin or other grafts around the new urethral canal for repair may reduce the occurrence of urinary leakage.^16^ In most clinical studies, priority is given to how to increase the success rate of surgery. However, few studies have been carried out on how to reduce the corresponding risk after surgery, and how to early identify and timely manage urethral stricture. Uroflowmetry, as a simple and non-invasive method to detect asymptomatic urethral stricture, is crucial for evaluating the postoperative outcome of hypospadias surgery and for timely detection of urethral strictures.^17^ There is a certain inevitable relationship between abnormal urine flow and urethral stricture.

It is considered that^18^ in 50% of hypospadias cases, abnormal urine flow is a risk warning of urethral stricture after surgery for hypospadias. Preoperative and postoperative urine flow assessment is of great significance. Qmax is a simple and effective method to evaluate urethral stricture.^19^ If the urine flow rate decreases, the possibility of urethral stricture should be suspected. Huang et al.^20^ believed that the combination of measurement of residual urine and measurement of urine flow rate can really help to exclude the cause of urethral obstruction. Only when the urine flow rate is obviously reduced and the residual urine is obviously increased can the cause of obstruction be considered. On the contrary, low urine flow rate and residual urine are caused by abnormal bladder function.

### Limitations

It includes a small sample size is included and the follow-up time is still short. Since the complications of hypospadias surgery are mostly late complications, it is particularly important to extend the follow-up time even to adulthood. In our future study, more cases will be included and the follow-up time will be extended, so as to further objectively evaluate the long-term effect and clinical significance of microscopic surgery for penile hypospadias.

## CONCLUSION

Microscopic surgery is a method with significant clinical value in the treatment of penile hypospadias, boasting benefits such as a high efficacy, less bleeding, shorter hospital stays, lower incidence of postoperative complications, and significantly improved urodynamics compared to conventional surgery.

### Authors’ Contributions:

**SG and WZ** designed this study and prepared this manuscript, and are responsible and accountable for the accuracy or integrity of the work.

**XS and BZ** collected and analyzed clinical data.

**JW** participated in acquisition, analysis, or interpretation of data and draft the manuscript.
